# The role of ^18^F-FDG PET/CT in primary cutaneous lymphoma: an educational review

**DOI:** 10.1007/s12149-023-01830-3

**Published:** 2023-04-24

**Authors:** Elysia O. McDonald, Amir A. Amanullah, Peter Sang Uk Park, William Song, Thomas J. Werner, Abass Alavi, Mona-Elisabeth Revheim

**Affiliations:** 1 Drexel University College of Medicine, Philadelphia, PA USA; 2 Temple University Lewis Katz School of Medicine, Philadelphia, PA USA; 3 University of Pennsylvania Perelman School of Medicine, Philadelphia, PA USA; 4 Department of Radiology, University of Pennsylvania Hospital, Philadelphia, PA USA; 5 The Intervention Center, Division of Technology and Innovation, Oslo University Hospital, Oslo, Norway; 6 Division of Radiology and Nuclear Medicine, Oslo University Hospital, Oslo, Norway; 7 Faculty of Medicine, Institute of Clinical Medicine, University of Oslo, Oslo, Norway

**Keywords:** Positron emission tomography, Primary cutaneous lymphoma, Fluorine-18-fluorodeoxyglucose, Nonattenuated PET, NAC

## Abstract

**Introduction:**

Primary cutaneous lymphoma (PCL) is a cutaneous non-Hodgkin’s lymphoma that originates in the skin and lacks extracutaneous spread upon initial diagnosis. The clinical management of secondary cutaneous lymphomas is different from that of PCLs, and earlier detection is associated with better prognosis. Accurate staging is necessary to determine the extent of disease and to choose the appropriate treatment. The aim of this review is to investigate the current and potential roles of ^18^F- fluorodeoxyglucose positron emission tomography–computed tomography (^18^F-FDG PET/CT) in the diagnosis, staging, and monitoring of PCLs.

**Methods:**

A focused review of the scientific literature was performed using inclusion criteria to filter results pertaining to human clinical studies performed between 2015 and 2021 that analyzed cutaneous PCL lesions on ^18^F PET/CT imaging.

**Results & Conclusion:**

A review of 9 clinical studies published after 2015 concluded that ^18^F-FDG PET/CT is highly sensitive and specific for aggressive PCLs and proved valuable for identifying extracutaneous disease. These studies found ^18^F-FDG PET/CT highly useful for guiding lymph node biopsy and that imaging results influenced therapeutic decision in many cases. These studies also predominantly concluded that ^18^F-FDG PET/CT is more sensitive than computed tomography (CT) alone for detection of subcutaneous PCL lesions. Routine revision of nonattenuation-corrected (NAC) PET images may improve the sensitivity of ^18^F-FDG PET/CT for detection of indolent cutaneous lesions and may expand the potential uses of ^18^F-FDG PET/CT in the clinic*.* Furthermore, calculating a global disease score from ^18^F-FDG PET/CT at every follow-up visit may simplify assessment of disease progression in the early clinical stages, as well as predict the prognosis of disease in patients with PCL.

## Background and Introduction

Primary cutaneous lymphoma (PCL) is a cutaneous non-Hodgkin’s lymphoma (NHL) that originates in the skin and lacks extracutaneous spread upon initial diagnosis. PCLs do not include lymphomas that secondarily spread to the skin. PCL has a wide variety of subtypes that differ in their clinical presentation prognosis. Indolent-behaving PCLs are slow-growing and less likely to metastasize; therefore, treatment is less invasive and involves primarily monitoring. High-grade PCLs, on the other hand, are more rapid in growth and dissemination and require more aggressive interventions. Conventional imaging techniques such as radiography and ultrasound may be used to screen for clinically indolent PCL. It is recommended that individuals with more aggressive-appearing PCL on physical exam undergo ^18^F- fluorodeoxyglucose positron emission tomography (^18^F-FDG PET) combined with computed tomography (CT) imaging to define extent of disease[1]. Treatment options and recommendations depend on both the stage and type of PCL; therefore, it is very important to accurately stage this disease by synthesizing key histologic and diagnostic imaging findings.

^18^F-FDG PET/CT is an imaging technology that is more specific, as well as sensitive, for detecting both cutaneous and extracutaneous PCL lesions compared to other imaging modalities used in clinical practice. Recent changes in World Health Organization–European Organization for Research and Treatment of Cancer (WHO-EORTC) protocol for staging and monitoring PCL have highlighted ^18^F-FDG PET/CT’s value as an imaging modality for staging and monitoring aggressive disease due to its unrivaled sensitivity and specificity. This review aims to explore the impact of ^18^F-FDG PET/CT in classifying, diagnosing, staging, and monitoring PCL.

### Incidence and classification

**Fig. 1:**
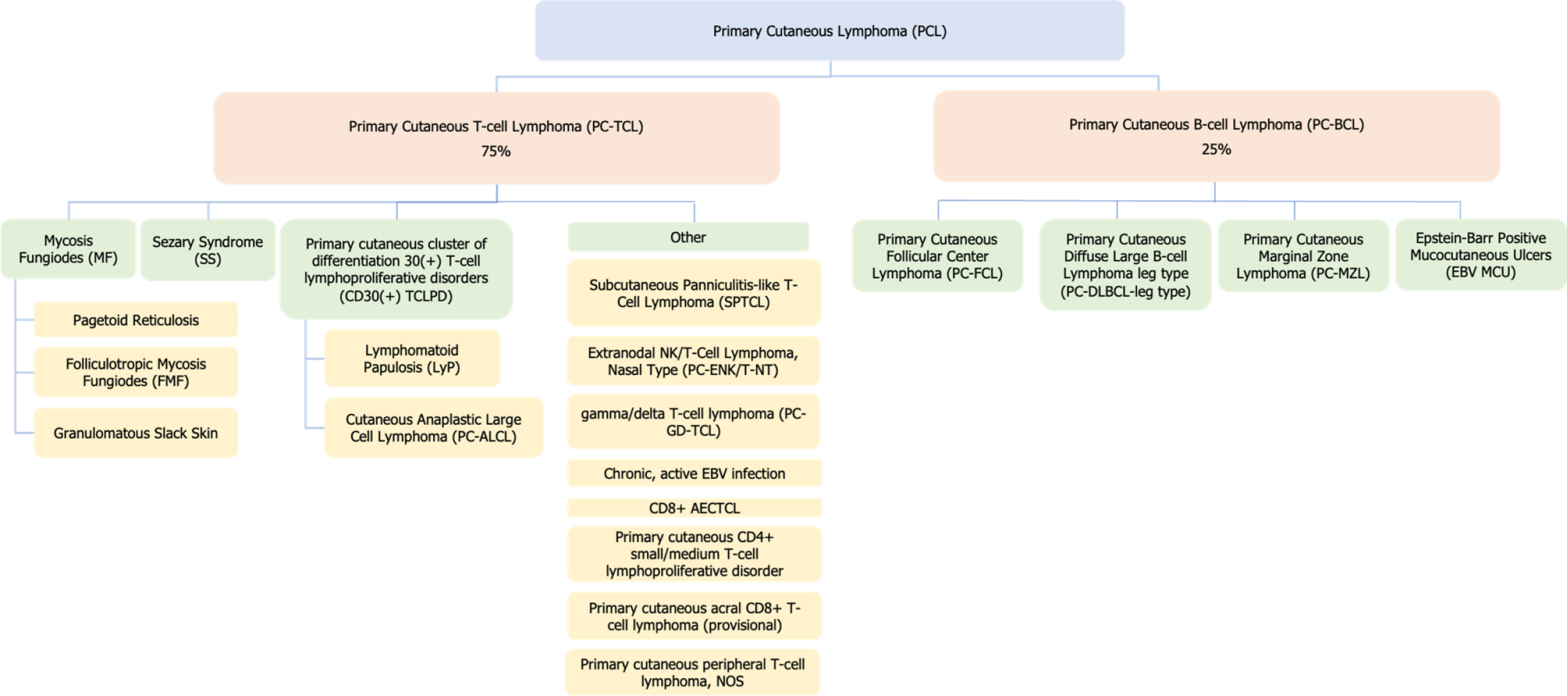
Subclassifications of primary cutaneous lymphomas. PCLs are classified by *T*-cell or *B*-cell lineage and subdivided according to clinical presentation, histopathology bimolecular profile and prognosis. PC-TCLs comprise roughly 75% of all PCLs, and PC-BCLs comprise the remaining 25%. Information courtesy of [2]

Lymphomas are subclassified into two broad categories: Hodgkin’s lymphoma (HL) and NHL. As of 2018, the WHO-EORTC consensus classification has been used as the gold standard for diagnosis and classification of primary cutaneous lymphomas [2]. PCL is the second most prevalent extra nodal NHL, occurring in 1 out of every 100,000 individuals in the United States. PCLs are classified depending on their specific cell lineage, i.e., *T*-cell or *B*-cell origin. Primary cutaneous *T*-cell lymphomas (PC-TCL) comprise roughly 75% of all PCLs, and primary cutaneous B-cell lymphomas (PC-BCL) comprise the remaining 25% (Fig. 1). Furthermore, PC-TCL and PC-BCL are each subdivided according to clinical manifestation, histopathology, biomolecular profile, and prognosis (Table 1). Fluorescence-activated cell sorting (FACS) is used to determine specific surface antigens present in cutaneous lymphoma cells and further classify these cells by their clonality.

**Table 1:** PCLs are broadly categorized into *T*-cell and *B*-cell subtypes. PC-TCLs generally display more aggressive behavior with poorer 5-year survival rates. PC-BCLs tend to be indolent, slow-growing and have more favorable 5-year survival rates

Cutaneous *T*-cell lymphomas	Frequency (%)	5-y DSS (%)	Behavior
Mycosis fungoides, early stage	39	88	Indolent
MF Variants			
Folliculotropic MF	5	75	Indolent
Pagetoid reticulosis	< 1	100	Indolent
Granulomatous slack skin	< 1	100	Indolent
Sézary syndrome	2	36	Aggressive
Adult *T*-cell leukemia/lymphoma	< 1	NDA	
Primary cutaneous CD30( +) *T*-cell lymphoproliferative disorders			
Primary Cutaneous Anaplastic Large-Cell Lymphoma	8	95	Indolent
Lymphomatoid Papulosis	12	99	Indolent
Subcutaneous panniculitis-like *T*-cell lymphoma	1	87	Indolent
Extranodal NK/T-cell lymphoma, nasal type	< 1	16	Aggressive
Chronic active EBV infection	< 1	NDA	
Primary cutaneous peripheral *T*-cell lymphoma, rare subtypes			
Primary cutaneous gamma/delta *T*-cell lymphoma	< 1	11	Aggressive
CD8 + AECTCL (provisional)	< 1	31	Aggressive
Primary cutaneous CD4 + small/medium *T*-cell lymphoproliferative disorder	6	100	Indolent
Primary cutaneous acral CD8 + *T*-cell lymphoma (provisional)	< 1	100	Indolent
Primary cutaneous peripheral *T*-cell lymphoma, not otherwise specified	2	15	Aggressive
Cutaneous *B*-cell Lymphomas			
Primary cutaneous marginal zone lymphoma	9	99	Indolent
Primary cutaneous follicle center lymphoma	12	95	Indolent
Primary cutaneous diffuse large *B*-cell lymphoma, *leg type*	4	56	Intermediate
EBV + mucocutaneous ulcer	< 1	100	Indolent
Intravascular large *B*-cell lymphoma	< 1	72	Indolent

*NDA* no data available, *5-y DSS* 5-year disease =-specific survival, Frequency and Prognosis of Primary Cutaneous skin lymphomas included in the WHO-EORTC classification, WHO-EORTC Classification [1, 2, 4]

Mycosis fungoides (MF) is the most prevalent PCL, accounting for 60% of all PC-TCLs, and 50% of all PCLs. Variants of MF are distinguished by their unique clinical presentations and histopathologic samples. Some of these variants include pagetoid reticulosis, folliculotropic MF (FMF), and granulomatous slack skin [2–4].

Sézary Syndrome is a rare PC-TCL subtype in which identification of peripheral blood involvement is necessary to differentiate it from other PC-TCL subtypes. Primary cutaneous cluster of differentiation (CD) 30( +) *T*-cell lymphoproliferative disorders (CD30( +) TCLPD) are the second most commonly occurring cutaneous T-cell lymphoma subtype as they contain 25% of all PC-TCLs. CD30( +) variants include lymphomatoid papulosis (LyP) and primary cutaneous anaplastic large-cell lymphoma (PC-ALCL). PC-TCL subtypes other than MF, Sézary syndrome, and CD30( +) TCLPD comprise less than 10% of PC-TCLs (Table 1). The latter include subcutaneous panniculitis-like T-cell lymphoma (SPTCL) and primary cutaneous extranodal NK/*T*-cell lymphoma, nasal type (PC-ENK/T-NT) [2].

Primary cutaneous B-cell lymphomas are divided into 4 subtypes: primary cutaneous follicular center lymphoma (PC-FCL), primary cutaneous diffuse large B-cell lymphoma *leg type* (PC-DLBCL-leg type), primary cutaneous marginal zone lymphoma (PC-MZL), and Epstein–Barr positive mucocutaneous ulcers (EBV MCU) (Table 1, Fig. 1). EBV MCU lymphomas comprise less than 1% of all PC-BCLs [1, 2].

### Diagnostic methods and staging

**Fig. 2:**
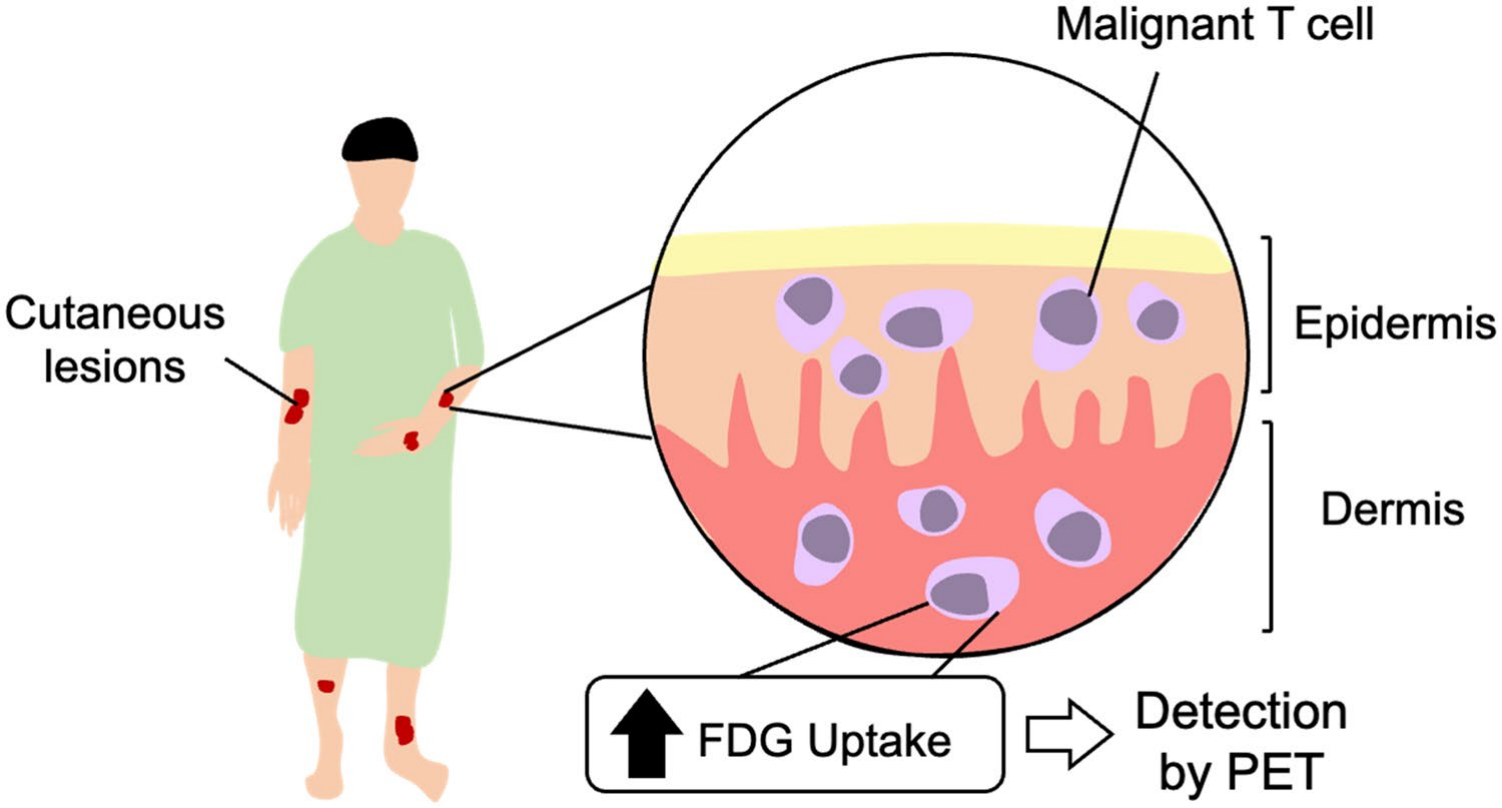
Schematic diagram of ^18^F-FDG PET in assessing cutaneous lesions of primary cutaneous lymphoma. Malignant *T*-cells exhibit increased metabolic activity and greater glucose uptake, which can be detected using ^18^F-FDG PET

The initial evaluation of all PCLs involves a physical examination with skin biopsy and blood draw for smear analysis. A clinician will look for presence of cutaneous lesions, such as erythroderma, plaques, and induration during the physical exam. PCL subtypes can be characterized by lesion location (e.g., trunk, extremities, face, etc.) and the presence of ulceraction. B-symptoms such as night sweats, fever greater than 38˚ Celsius/100.4˚ Fahrenheit for at least one week, and a minimum 10% weight loss within the past six months is suggestive of extracutaneous spread of disease [5]. The lymph nodes (e.g., cervical, inguinal, axillary), liver and spleen, are palpated during the physical exam to determine presence of swelling, as these are common sites to which PCL initially metastasizes. Enlarged lymph nodes or unusual cutaneous lesions should be biopsied. The WHO-EORTC recommends a bone marrow biopsy for patients with intermediate or aggressive subtypes of PCL. ^18^F-FDG PET/CT imaging should be performed for any patient with suspected extracutaneous disease, or with a predominantly subcutaneous-presenting PCL (Fig. 2) [6].

Diagnostic blood tests for PCLs include a complete blood count with differential, comprehensive metabolic panel including lactate dehydrogenase (LDH) levels, electrolytes, liver enzymes, and creatinine. Both elevated erythrocyte sedimentation rate and c-reactive protein serum levels are associated with poor prognosis in PCL patients [5]. Patients with erythroderma require peripheral blood taken for smear analysis to determine possible presence of characteristic Sézary cells [1, 2].

Genetic and biomolecular testing to identify cellular markers using FACS is performed to determine the CD4:CD8 cell ratio and PCL clonal subtype (CD3 + /CD7, CD3 + /CD26). Specific CD markers are useful in identifying whether the cell originates from the *B*-cell or *T*-cell lineage. This information is used for “blood staging” to determine total tumor burden of the blood [7].

PCL staging uses the tumor–node–metastasis–blood (TNMB) classification system [7, 8] which considers features of disease regarding skin lesions, lymph nodes, visceral organ involvement, and blood tumor burden. A revised TNMB classification guides the clinical staging of MF and Sézary syndrome (Table 2A, 2B) [9], while a separate TNM system is used to classify and stage non-MF or non-Sézary syndrome primary cutaneous lymphomas (Table 2C) [7, 10]. The “T” category describes the severity of skin lesions and is subdivided into T1 through T4. The “N” category describes lymph node involvement, and “M” describes metastasis and if the disease has spread to regional lymph nodes or extracutaneous organs. “B” describes what the “burden” or concentration of these cells is in the blood [1].

**Table 2:** Clinical staging of MF/Sézary primary cutaneous lymphomas is performed using the TNMB classification (2A, 2B). The clinical presentation of non-MF/Sézary PCLs is significantly different from that of MF/Sézary PCLs, therefore the TNM classification, instead of the TNMB, is used to aid clinical staging of non-MF/Sézary PCLs (2C)

A. TNMB classification of MF/Sézary syndrome	
(T) Skin	
T1	Limited patch or plaque < 10% total skin surface area
T2	Generalized patch or plaque > 10% total skin surface area
T3	Tumor(s)
T4	Erythroderma
(N) Lymph Node	
N0	No clinically abnormal peripheral lymph nodes
N1	Clinically abnormal peripheral nodes; histologically normal
N2	Clinically abnormal peripheral nodes; histologically involved (uneffaced nodal architecture)
N3	Clinically abnormal peripheral nodes; histologically involved (partially effaced nodal architecture)
Nx	Clinically abnormal peripheral nodes; not histological confirmation
(M) Viscera	
M0	No visceral involvement
M1	Visceral involvement
(B) Blood	
B0	No circulating atypical (Sézary) cells (or < 5% of lymphocytes)
B1	Low blood tumor burden (> or equal to 5% lymphocytes are Sézary cells)
B2	High blood tumor burden (> or equal to 100/μL Sézary cells and positive clone)

B. Clinical staging of MF/Sézary using TNMB classification				
IA	T1	N0	M0	B0-1
IB	T2	N0	M0	B0-1
IIA	T1-2	N1-2	M0	B0-1
IIB	T3	N0-2	M0	B0-1
III	T4	N0-2	M0	B0-1
IVA1	T1-4	N0-2	M0	B2
IVA2	T1-4	N3	M0	B0-2
IVB	T1-4	N0-3	M1	B0-2

C. TNM classification of non-MF/Sézary primary cutaneous lymphomas	
(T) Skin	
T1	Solitary skin lesion
	T1a: lesion size < 5 cm diameter
	T1b: lesion size < 5 cm diameter
T2	Multiple skin lesions confined to 1 body region or 2 contiguous body regions
	T2a: all-disease-encompassing in a < 15 cm diameter circular area
	T2b: all-disease-encompassing in a > 15- and < 30 cm diameter circular area
	T2c: all-disease-encompassing in a > 30 cm diameter circular area
T3	Generalized skin involvement
	T3a: multiple lesions involving 2 noncontiguous body regions
	T3b: multiple lesions involving > or equal to 3 body regions
(N) Lymph node	
N0	No clinical or pathologic lymph node involvement
N1	1 peripheral lymph node involved, drains area of diseased skin
N2	2 or more peripheral lymph nodes in region of diseased skin OR involvement of 1 or more lymph nodes not in region of affected skin
N3	Central lymph nodes involved
(M)Viscera	
M0	No evidence of extracutaneous non-lymph node disease
M1	Extracutaneous non-lymph node disease is present

PC-BCLs tend to be indolent, slow-growing and have more favorable 5-year survival rates. *NDA* no data available, *5-y DSS* 5-year disease specific survival

Courtesy of the American Society of Hematology and WHO-EORTC [1, 9], Courtesy of Kim 2007 [10]

### TNMB staging of MF and Sézary syndrome primary cutaneous lymphomas

MF and Sézary syndrome must be staged and treated differently than other PCLs because of their unique presentations and progressions. Disease response in the skin is assessed using mSWAT score [11]. A baseline CT is recommended for initial diagnosis of all MF and Sézary syndrome lymphomas, but subsequent scans are generally not recommended in patients with early disease unless nodal spread is suspected. If nodal or visceral spread of disease is suspected, baseline CT should be performed, along with interim imaging to assess early response to treatment, and imaging at the end of treatment to determine success. Stage 1 of MF or Sézary syndrome lymphoma is limited to the skin as patches or plaques. Substages can be classified depending on presence of B-symptoms, lymph node adenopathy, lesion characteristics and peripheral blood involvement.

### TNM staging of non-MF/Sézary syndrome primary cutaneous lymphomas

Non-MF and non-Sézary syndrome PCLs are staged using different criteria than those used to stage MF or Sézary syndrome PCLs. The International Society for Cutaneous Lymphomas (ISL) and EORTC has proposed a TNM system of staging that excludes using B-symptoms as a criterion (Table 2C) [10]. This system has a subclassification system using “a”, “b” and “c” to denote varying lesion sizes. “T1” indicates a solitary lesion and is subsequently broken down into two categories: “T1a” lesion < 5 cm in size, “T1b” > 5 cm in size. T2 indicates multiple lesions affecting one or two contiguous body regions and T3 indicates the disease affects the skin diffusely. “N” refers to nodal involvement, with N0 stage being used to classify cancers that lack nodal involvement. “M” indicates whether extracutaneous disease is present.

For all PCL subtypes, the imaging modality chosen for staging is determined by the staging score; T1 skin involvement with B0 blood burden suggests that only a chest radiograph is needed to scan for visceral organ disease. CT with contrast imaging of the chest, pelvis and abdomen is recommended for all higher TNM/TNMB disease scores. As of 2018, the European Society for Medical Oncology (ESMO) strongly recommends ^18^F-FDG PET/CT imaging for aggressive cutaneous lymphomas, late-stage lymphomas, or lymphomas with signs of extracutaneous disease [1]. TNM/TNMB classifications are only used for staging disease and are not valid predictors of patient prognosis.

### Managing indolent versus aggressive PCL

After diagnosis of PCL, subsequent steps in management of disease vary based on whether a cutaneous lymphoma is classified as “indolent” or “aggressive” (Table 1) [2]. Indolent lymphomas in the early stage may be imaged with conventional methods such as a chest X-ray or ultrasound of the abdomen and superficial lymph nodes [12]. CT scan is the recommended imaging modality to assess higher stage PCL lesions which are not ^18^F-FDG-avid. Whole-body ^18^F-FDG-PET/CT is strongly recommended for more aggressive or late-stage cutaneous lymphomas such as PC-DLBCL-leg type, PC-FCL, and Sézary syndrome [12]. Staging of disease should be performed annually at minimum to monitor progress of disease. Clinical stage of tumor at time of diagnosis is the most predictive factor when determining disease prognosis. A widely agreed upon, standardized protocol for staging PCL would increase clarity of communication amongst clinicians and more effective collaborative efforts may enable more accurate staging and treatment of PCLs [13]. For PCL, the effectiveness of interventions is highly dependent on appropriate staging of disease; therefore, improving the accuracy of staging with a standardized protocol would, in theory, lead to more effective application of interventions and improved disease outcomes.

The prognosis of all NHLs is predicted using the International Prognostic Index (IPI), which accounts for patient age, LDH levels, location of lesions, and extent of skin involvement [13–15]. Subtypes of PCL have individualized criteria to generate a prognostic score specific for that subtype of disease [12]. The IPI score, used to determine treatment in aggressive primary cutaneous NHL subtypes such as PC-DLBCL-*leg type* and primary cutaneous peripheral T-cell lymphoma, considers updated Ann Arbor criteria, disease stage, serum LDH, hemoglobin, patient age, and patient “performance status” [15–17].

## Methods

Our search determined that nine new studies investigating the role of ^18^F-FDG PET/CT in PCLs have been published between 2015 and 2021 (Table 3) [6, 12, 18–24]. These studies were screened and selected utilizing Google scholar search engine. An advanced Google Scholar search for articles published between the years 2015 and 2021 that contained the exact phrase “primary cutaneous lymphoma” anywhere in the article yielded 885 results. These 885 results contained review papers, case studies and clinical trials used for the results and discussion section of this review paper.

**Table 3:** Studies reviewed reflect the major updates to the field of ^18^F-FDG PET/CT in PCL since 2014

Study, year	Design	Subject #	Results	Conclusions
[6]	Retrospective analysis of baseline and post-treatment ^18^F-FDG PET/CT scans	11 patients with subcutaneous panniculitis-like T-cell lymphoma	At initial PET/CT scans, 5/11 patients had extracutaneous non-lymph node lesions with SUVmax of 5.6 ± 2.8 on ^18^F-FDG PET/CT. Follow-up ^18^F-FDG PET/CT scans in 6 patients revealed complete remission of the disease in 2, partial remission in 3, and progressive disease in 1 (Fig. 3)	The superiority of ^18^F-FDG PET/CT over CT alone at detecting subcutaneous lesions makes it a useful tool for mapping out extent of disease, guiding biopsy, diagnosing, staging, and evaluating treatment response in patients with ^18^F-FDG-avid PCLs like SPTCL
[12]	Retrospective analysis of baseline and post-treatment ^18^F-FDG PET/CT scans	Retrospective analysis of the ^18^F-FDG PET/CT for 17 patients diagnosed with PCL; 8 patients had PC-FCL and 9 had PC-MZL	^18^F-FDG PET/CT detected cutaneous lesions in 4/8 of the PC-FCL patients, and in 5/9 of the PC-MZL patients	^18^F-FDG PET/CT is more sensitive than CT for detecting cutaneous lesions and allows for visualization of lesions not identified on physical exam. However, PC-BCL lesions are not ^18^F-FDG avid in a significant proportion of patients
[18]	Prospective study analysis of baseline ^18^F-FDG PET/CT scans compared to baseline CT scans	A total of 18 cases with non-MF/SS PCL, were analyzed in this study. 13 of these were T-cell or NK PCL, 5 of these were PC-BCL, and 1 was a "precursor hematologic neoplasm"	Non-MF/SS PCL in this study: PC-MZL, diffuse large B-cell PCL, anaplastic large-cell PCL, PC-ENK/T-NT and SPTCL. The diagnostic sensitivity of CT and PET/CT scans in the diagnosis of primary skin lesions was 82.4% (14/17) and 100% (17/17), respectively. 3/18 cases of cutaneous PCL were missed by CT, while ^18^F-FDG PET was positive for detection of skin lesions in 18/18 cases (Fig. 4)	Compared to CT alone, ^18^F-FDG PET/CT is more sensitive for detection of malignant skin lesions. Subcutaneous skin lesions are easily missed by CT. ^18^F-FDG PET/CT should be used over CT alone for detection of subcutaneous-presenting B-cell and T-cell PCL, especially SPTCL and PC-ENK/T-NT
[19]	Retrospective analysis of the baseline and post-treatment ^18^F-FDG PET/CT scans	20 patients with MZL had subcutaneous lesions identified on ^18^F-FDG PET/CT	20 patients with subcutaneous MZL verified by biopsy (Fig. 5). Subcutaneous MZL lesions are easier to detect on ^18^F-FDG PET than on CT scans. The detection of subcutaneous MZL lesions changed the disease stage in 8 patients (40%) and resulted in a therapeutic decision change in 2 patients (10%)	^18^F-FDG PET/CT analysis may help in the detection of indolent subcutaneous MZL with low ^18^F-FDG avidity and improve diagnostic sensitivity, staging, and treatment decisions
[24]	Retrospective analysis of baseline ^18^F-FDG PET/CT scans	33 patients with PC-BCL: 26 (79%) had small-cell PC-BCL (18 marginal zone, 8 follicle center lymphoma) and 7 (21%) had large-cell PC-BCL (3 follicle center, 3 leg type, 1 indeterminate)	^18^F-FDG PET/CT detected skin lesions in 3 of 26 patients (12%) with small-cell PC-BCL as compared to 6 of 7 patients with large-cell PC-BLC (86%), a 7.4-fold higher detection rate (95% confidence interval, 2.4–22, *P* = 0.004). PET-positive lesions were larger size (*P* < 0.001) and a higher Ki-67 proliferation index (*P* < 0.001). PET/CT detected 100% of PC-LCL-LT and diffuse large-cell lymphoma, which are considered intermediate-aggressive types, and only 11% of PC-MZLs and 27% of PC-FCLs, which are indolent types	The sensitivity of ^18^F-FDG PET/CT for detecting cutaneous lesions is low for indolent B-cell PCLs like PC-MZL and PC-FCL, and high for aggressive PC-BCL like PC-DLBCL-leg type
[23]	Retrospective analysis of CXR, HRUS and ^18^F-FDG PET/CT images	41 patients diagnosed with PCL (33 *B-*cell and 8 *T-*cell lymphomas) had imaging performed during initial diagnosis and or staging follow-up visits. ^18^F-FDG PET/CT scan was performed in 13 cases	Whole-body ^18^F-FDG PET/CT imaging detected cutaneous lesions in 12/13 patients; the missed lesion was a B-cell PCL. PET/CT displayed increased ^18^F-FDG uptake in 92.3% of cutaneous lesions. HRUS was useful for visualizing extent of tumor infiltration in the cutaneous layers and for characterizing vascularity, calcifications, and density of lesions	^18^F-FDG PET/CT is the recommended modality of imaging for staging and follow-up in both B-cell and T-cell PCLs
[21]	Retrospective analysis of ^18^F-FDG PET/CT scans	11 patients with PC-ALCL had both ^18^F-FDG PET/CT and CT baseline imaging performed. Results of ^18^F-FDG PET/CT compared with those of CT. Biopsy results served as a reference for the accuracy of PET and CT	The sensitivity of ^18^F-FDG-PET was 64% versus 18% for CT, demonstrating the added value of ^18^F-FDG-PET in initial staging for patients with PC-ALCL first presenting in the skin. In PC-ALCL, ^18^F-FDG-PET influences the therapeutic decision, except in T1aN0M0-classified patients	^18^F-FDG PET/CT is valuable for the initial staging of PC-ALCL because it is more sensitive than CT alone for detection of cutaneous and extracutaneous lesions. Earlier detection of PC-ALCL by PET/CT may impact therapeutic decisions
[20]	Retrospective analysis of baseline ^18^F-FDG PET/CT scans compared to CT and MRI, conventional staging methods	39 patients newly diagnosed with PC-ENK/T-NT imaged with ^18^F-FDG PET/CT, CT and MRI. Skin biopsies used to verify diagnosis	In the detection of malignant PC-ENK/T-NT skin lesions, ^18^F-FDG PET/CT detected 48/50 cutaneous and CT/MRI detected only 34 cutaneous lesions. ^18^F-FDG PET/CT is 96% sensitive and 98.6% specific, while CT/MRI are 68% sensitive and 97.9% specific (*P* < 0.001). ^18^F-FDG PET/CT staging was consistent with the final stage determination in 94.9% (37/39) of patients, whereas CT/MRI staging was correct in final stage determination in 74.4% (29/39) of patients (*P* = 0.025)	^18^F-FDG PET/CT scanning is more accurate than CT or MRI for the detection of cutaneous and extracutaneous lesions of PC-ENK/T-NT. ^18^F-FDG PET/CT is a superior tool for staging and evaluation of patient response to treatment
[22]	Retrospective analysis of ^18^F-FDG PET/CT scans prior to treatment	19 patients (mean age, 40.6 years; median age 41 years; 16 males and 3 females) diagnosed with mycosis fungoides and with risk of secondary LN involvement were included in the study	^18^F-FDG-avid cutaneous lesions visualized via PET/CT in 13 out of 19 patients. 4 patients underwent ^18^F-FDG PET/CT which enabled visualization of disease response to treatment in cutaneous lesions. (Fig. 8). ^18^F-FDG PET/CT is more sensitive than CT for detection of diseased LNs (Fig. 7A, B). Physical exam is superior to ^18^F-FDG PET/CT in the detection of thin, superficial cutaneous lesions; Review of NAC PET images improves detection of superficial cutaneous lesions	^18^F-FDG PET/CT is valuable for guiding biopsy of high-grade lesions that are not always visible on physical exam. Whole-body ^18^F-FDG PET/CT should be performed at initial staging of MF, and NAC PET images should always be evaluated

A nested search was performed to summarize recent clinical trial research focused on defining the role of ^18^F-FDG PET/CT in diagnosing, staging, and treating primary cutaneous lymphomas. This nested search for articles used advanced Google Scholar search engine to discover all articles published between 2015 and 2021 that contain the exact phrase “primary cutaneous lymphoma” AND at least one of the words “positron emission tomography” OR “PET CT” OR "positron emission tomography computed tomography" anywhere in their text. This search yielded 231 results. Of these results, only scientific studies composed of > nine human subjects, that described ^18^F-FDG PET/CT detection of cutaneous/subcutaneous PCL lesions, were included. After performing the literature search using the inclusion criteria as described, a total of nine articles remained and were included in this review.

## Results and discussion

### Other imaging modalities: compared to ^18^F-FDG PET/CT

**Fig. 3:**
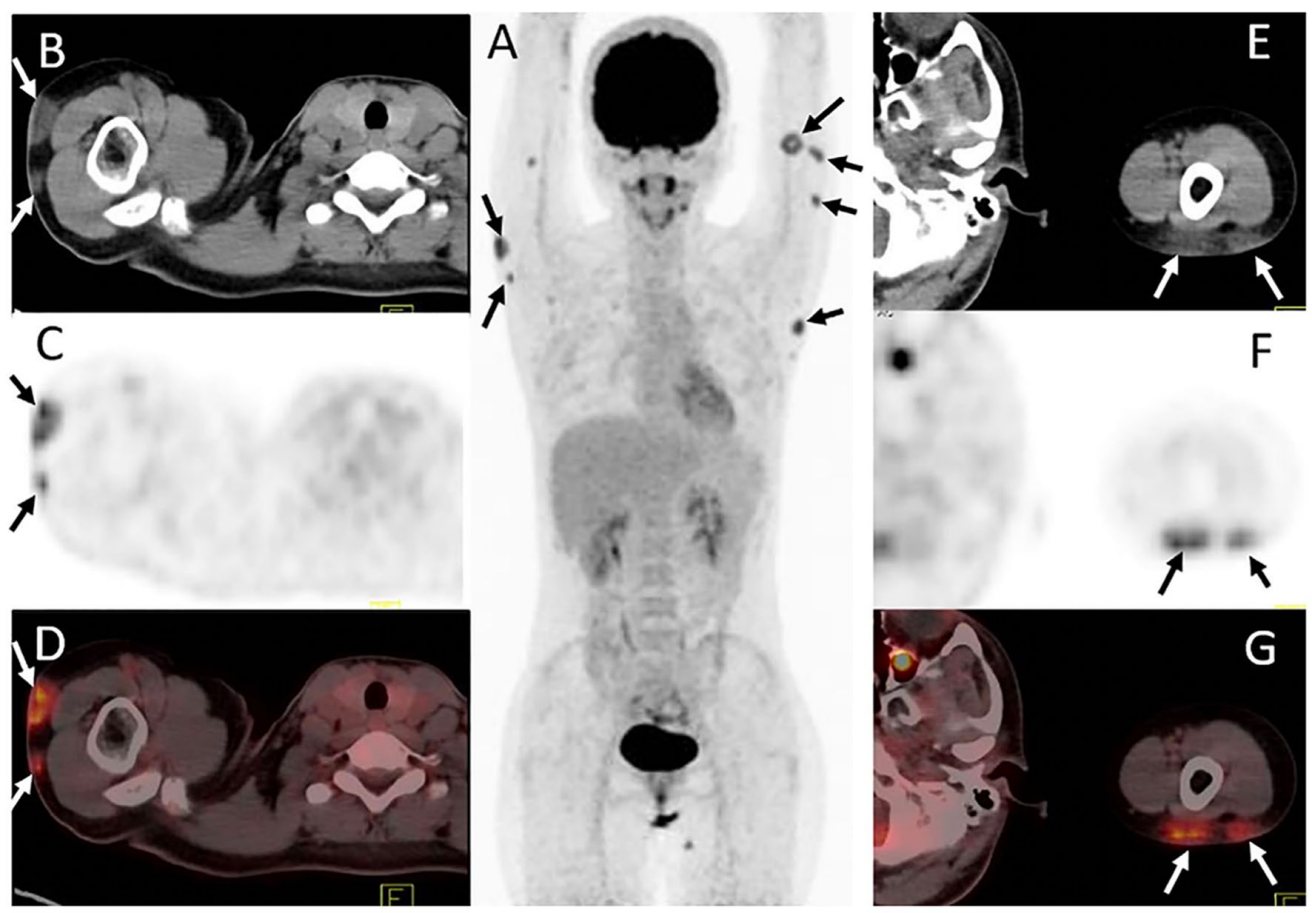
Subcutaneous SPTCL lesions are easy to miss on CT and visualization is significantly improved with ^18^F-FDG PET/CT. A 24-year-old female with subcutaneous panniculitis-like *T*-cell lymphoma located in the upper arms (black arrows). ^18^F-FDG PET/CT 3-dimensional maximum intensity projection image (**A**), axial CT (**B**, **E**) and axial fusion PET/CT (**D**, **G**) images reveal multiple subcutaneous lesions displaying high ^18^F-FDG uptake in the skin. Images courtesy of Jiang 2021 [6]

**Fig. 4:**
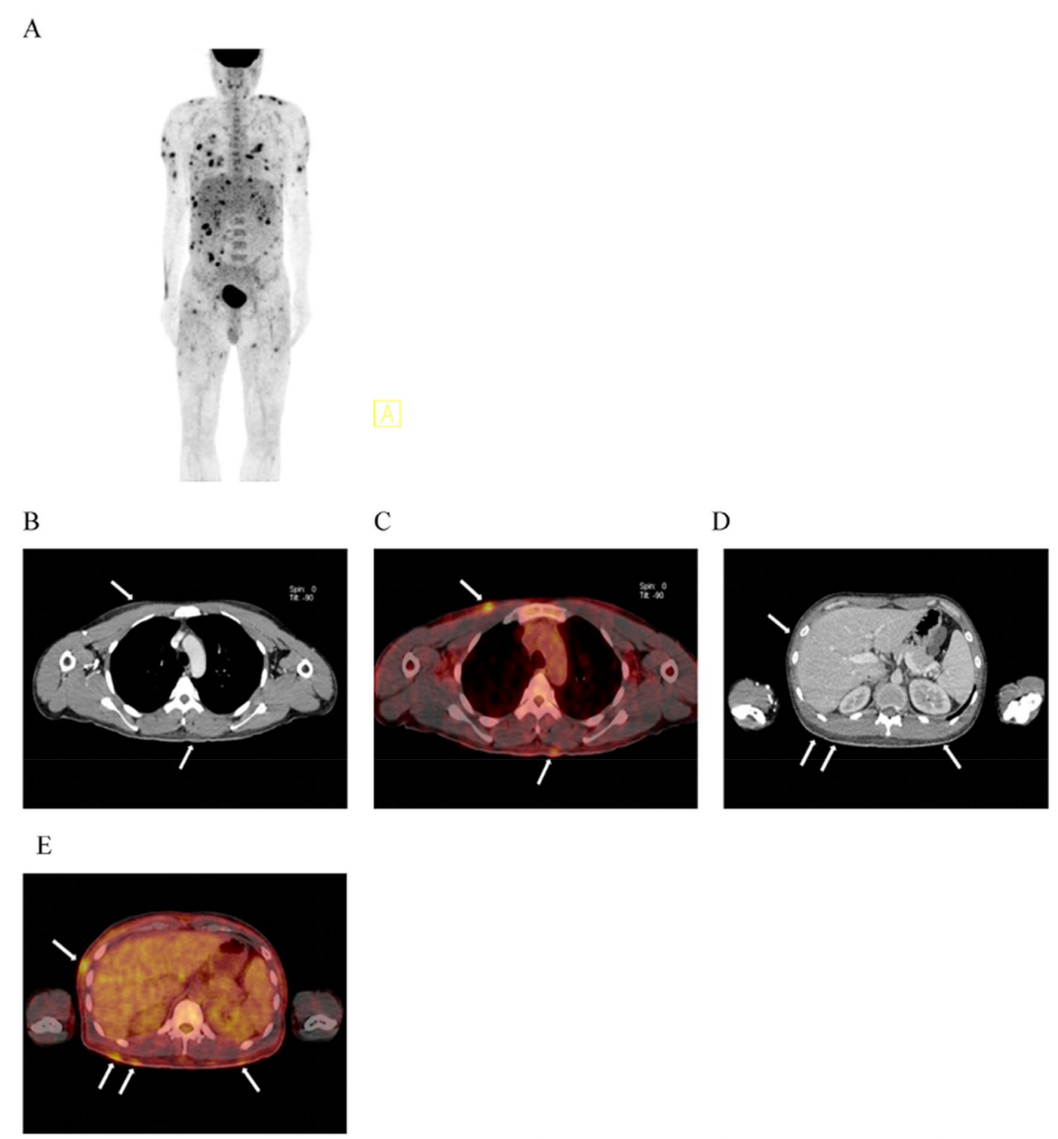
A 30-year-old man diagnosed with subcutaneous panniculitis-like *T*-cell lymphoma. ^18^F-FDG PET/CT three-dimensional maximum intensity projection image (**A**) and axial fused PET/CT (**C**, **E**) reveals multiple focal lesions displaying intense ^18^F-FDG uptake in the skin. Axial contrast-enhanced CT (**B**, **D**) failed to identify these lesions. Images courtesy of Dan 2015 [18]

**Fig. 5:**
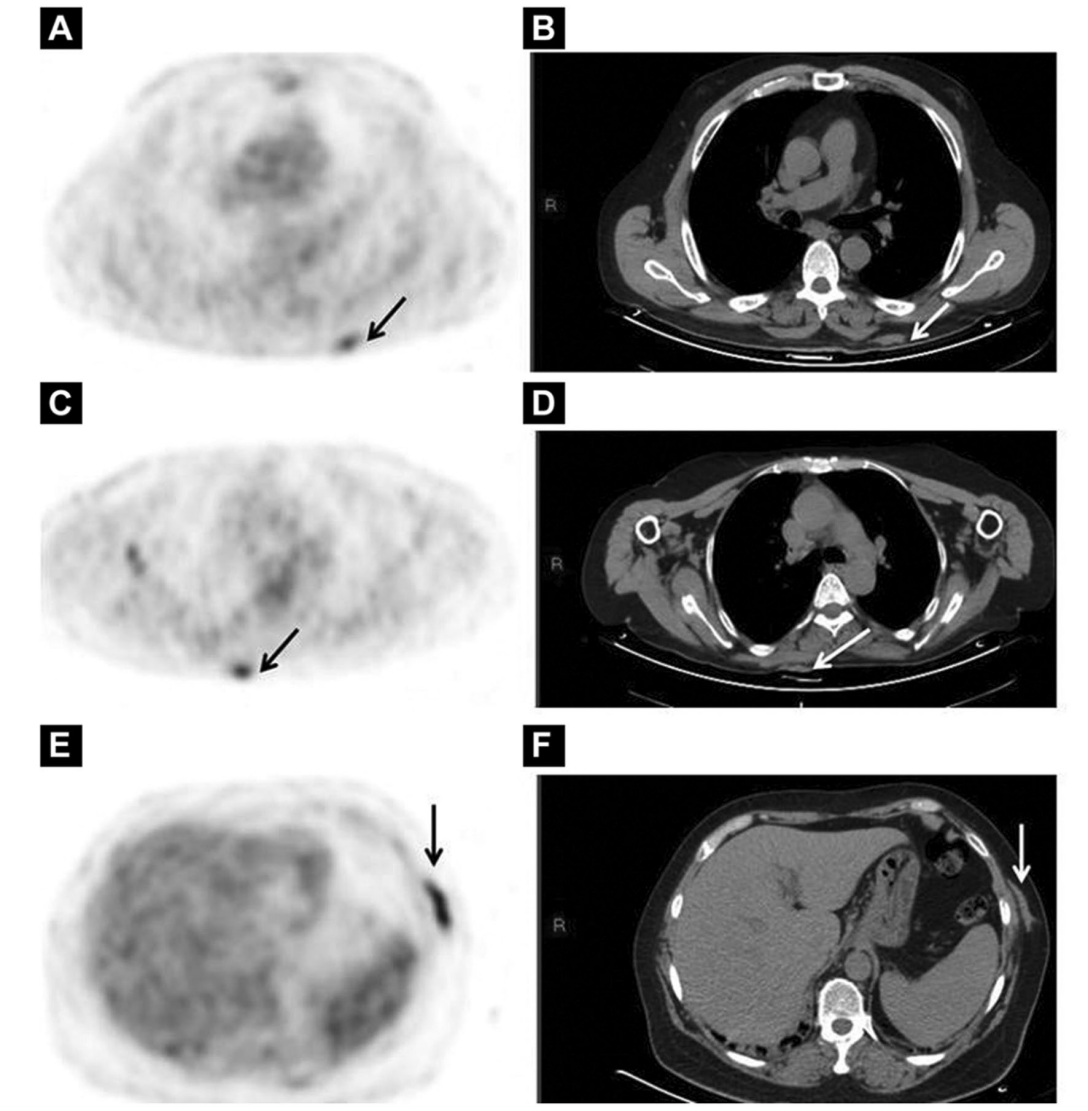
A patient with cutaneous marginal zone lymphoma, an indolent *B*-cell PCL. Subcutaneous lesions (white arrows) on axial CT (**B**, **D**, **F**) are visible but difficult to detect. These same lesions are more distinct and easier to identify on axial ^18^F-FDG PET/CT (**A**, **C**, **E**). This study provides evidence that ^18^F-FDG PET/CT may be superior to CT at detecting not only aggressive PCLs, but also indolent PCLs with subcutaneous presentation. Images courtesy of Davidson 2020 [19]

Currently, CT is the primary imaging modality used to detect PCL lesions. Although CT is highly sensitive for extracutaneous disease, it has shortcomings in scenarios which require detection of cutaneous malignant lesions. While contrast-CT may delineate lesions, it does not provide functional, metabolic information. This lack of functional data may lead the clinician to overlook small, yet aggressive lesions, subcutaneously presenting lesions such as SPTCL (Fig. 3 and Fig. 4) and PC-ENK/T-NT, and diseased lymph nodes that appear to be ‘normal’ in size [6, 18]. Lack of functional metabolic information is also why CT is prone to false-positive findings in enlarged but benign lymph nodes [25]. Some studies propose that ^18^F-FDG PET/CT is the preferred imaging modality for diagnosing and staging of PCLs due to its superior sensitivity to CT (Fig. 5) [18, 20, 21, 23].

In clinical practice, ^18^F-FDG PET/CT is not routinely used for disease staging in patients with PCL. Other imaging modalities such as radiography, ultrasound, CT, and magnetic resonance imaging (MRI) are used in combination with physicians’ physical assessment findings to fully stage PCL. ^18^F-FDG PET/CT is not sensitive for cutaneous lesions with low metabolic activity, which is commonly a characteristic of early-stage PCL and indolent subtypes of PCL [12, 19, 24, 26]. While ^18^F-FDG PET/CT has not been shown to effectively detect early-stage cutaneous PCL lesions, it is, however, very sensitive for highly metabolically active cutaneous and visceral PCL lesions in aggressively presenting late-stage disease (Fig. 2) [1]. Weighing the cost–benefit ratio of exposing patients to radiation plays an important part in assessment and treatment plan, as clinicians prefer to minimize radiation exposure in patients with limited or indolent disease. As result, until recently the role of ^18^F-FDG PET/CT imaging in PCL has been limited to patients with aggressive, extra nodal, or primarily subcutaneous disease [1]. However, the latest generations of whole-body PET/CT instruments have a sensitivity 15–68-fold higher than that of conventional PET/CT instruments, requiring lower doses of radiotracer. With whole-body PET imaging, the patient is exposed to far less radiation per image acquired, and the improved safety of this imaging modality broadens its potential clinical applications [27].

**Fig. 6:**
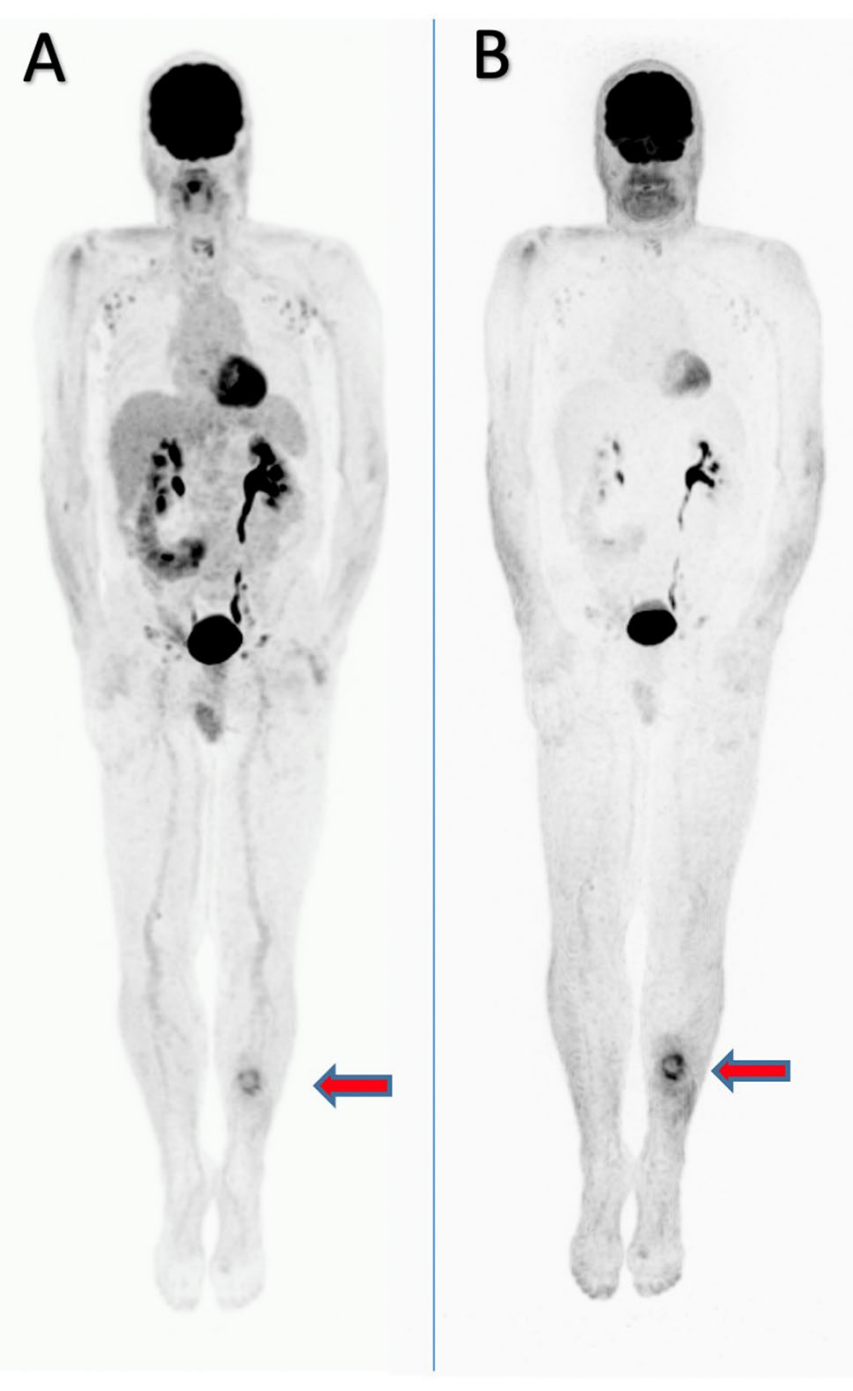
3D maximum intensity projection image of an adult diagnosed with PCL. Nonattenuation-corrected ^18^F-FDG PET images elucidate thin or indolent cutaneous PCL lesions that are often missed on attenuation-corrected ^18^F-FDG PET analysis. In (**A**), the attenuation-corrected ^18^F-FDG PET scan allows for faint visualization of a superficial cutaneous PCL lesion (red arrow) on the anterior left calf. Attenuation correction dampens ^18^F-FDG signal from superficial cutaneous lesions. In (**B**), the corresponding nonattenuation-corrected ^18^F-FDG PET scan of this patient displays the ^18^F-FDG uptake of this lesion (red arrow) more accurately, allowing for better characterization of its location and metabolic activity

To the authors’ knowledge, current methods of diagnosis have not investigated the impact that evaluation of nonattenuation-corrected (NAC) PET images may have on the sensitivity of ^18^F-FDG PET/CT for detecting cutaneous PCL lesions. NAC PET images are often disregarded; however, NAC PET images can provide invaluable information about superficial lesions and deserve more attention [22, 28–30]. As demonstrated in Fig. 6, NAC PET elucidates thinner or less metabolically active cutaneous lesions that are often missed in analysis using solely attenuation-corrected (AC) PET scans (Fig. 6). This is because of the low signal intensity of superficial lesions, which is often lost during the process of attenuation correction of images. It is recommended that nuclear medicine radiologists use the information provided by NAC PET, along with the information provided by AC ^18^F-FDG PET/CT images, as this would ensure that all cutaneous PCL lesions are accounted for [22, 28–30].

### The role of ^18^F-FDG PET/CT in diagnosis and staging

Stage of disease is the most important predictor of disease prognosis in PCL, and thus tumor staging is heavily used to guide the clinician’s generation of an individualized, patient-centered treatment plan. ^18^F-FDG PET/CT is sensitive and specific for aggressive PCLs, facilitating detection of extracutaneous disease. While ^18^F-FDG PET/CT influences PCL staging due to its sensitivity for disease manifestation in skin, lymph nodes and extracutaneous tissues, this imaging modality has no value in the pure classification of PCLs. PCLs are classified based on whether they are *T*-cell or *B*-cell in origin, and a skin biopsy is necessary for definitive classification (Table 1, Fig. 1).

In centers that have access to this state-of-the-art technology, ^18^F-FDG PET/CT is quickly becoming the preferred imaging modality for staging PCLs [31]. In 2018, the European Society for Medical Oncology (ESMO) published updated guidelines for the diagnosis and treatment of PCL [1]. Quantification of ^18^F-FDG uptake in malignant cells may allow clinicians to generate a global disease score to be used for monitoring PCL progression [32]. While ^18^F-FDG PET/CT has proven itself useful for the monitoring of disease progression in patients with PCL, significant debate persists regarding which imaging modality is best for predicting disease prognosis and monitoring PCL response to treatment. ^18^F-FDG PET/CT is proven to be very sensitive for extracutaneous spread of PCL, but it has been criticized for its poor sensitivity for indolent cutaneous PCL lesions [1, 21, 24]. However, in subtypes of PCL that display high tendency for metastasis, such as MF or PC-ALCL, it is argued that PET imaging should always be performed at initial staging to determine full extent of disease [21–23].

Several recent studies state that utilization of ^18^F-FDG PET/CT over CT alone would improve the accuracy of initial PCL staging at diagnosis. A study concluded that ^18^F-FDG PET/CT may increase accuracy of initial staging of PC-MZL, a B-cell PCL that is often missed on physical exam due to its tendency to present primarily subcutaneously. In this study, ^18^F-FDG PET/CT displayed superior sensitivity for detection of subcutaneous lesions as compared to CT alone (Fig. 5) [19]. A study by Liu et al. determined that ^18^F-FDG PET/CT is superior to CT or MRI in the initial staging of PC-ENK/T-NT. ^18^F-FDG PET/CT initial disease staging was more consistent with final staging (confirmed by biopsy) in 94.9% (37/39) of patients, while CT/MRI staging was consistent with final staging in only 74.4% (29/39) of cases [20]. Another study found that in patients with PC-ALCL, initial staging by ^18^F-FDG PET/CT was more accurate than staging by CT. The sensitivity of ^18^F-FDG PET/CT for cutaneous lesions was 64% as compared to 18% sensitivity for CT alone[21]. Moreover, a study of 18 patients with non-MF/SS PCL found that ^18^F-FDG PET/CT has higher sensitivity than CT alone for detection of primary skin lesions, and that CT alone is likely to miss subcutaneous PCL lesions such as SPTCL and PC-ENK/T-NT (Fig. 4). In a cohort of 18 patients, 3 cases of subcutaneous PCL were missed by CT alone; 100% of cases were identified by ^18^F-FDG PET/CT [18]. Overall, multiple recent studies state that initial staging of PCL is more accurate when performed via ^18^F-FDG PET/CT than by CT alone. This is true for both indolent and aggressive *B*-cell and *T*-cell PCLs.

### Guidance of biopsy

**Fig. 7:**
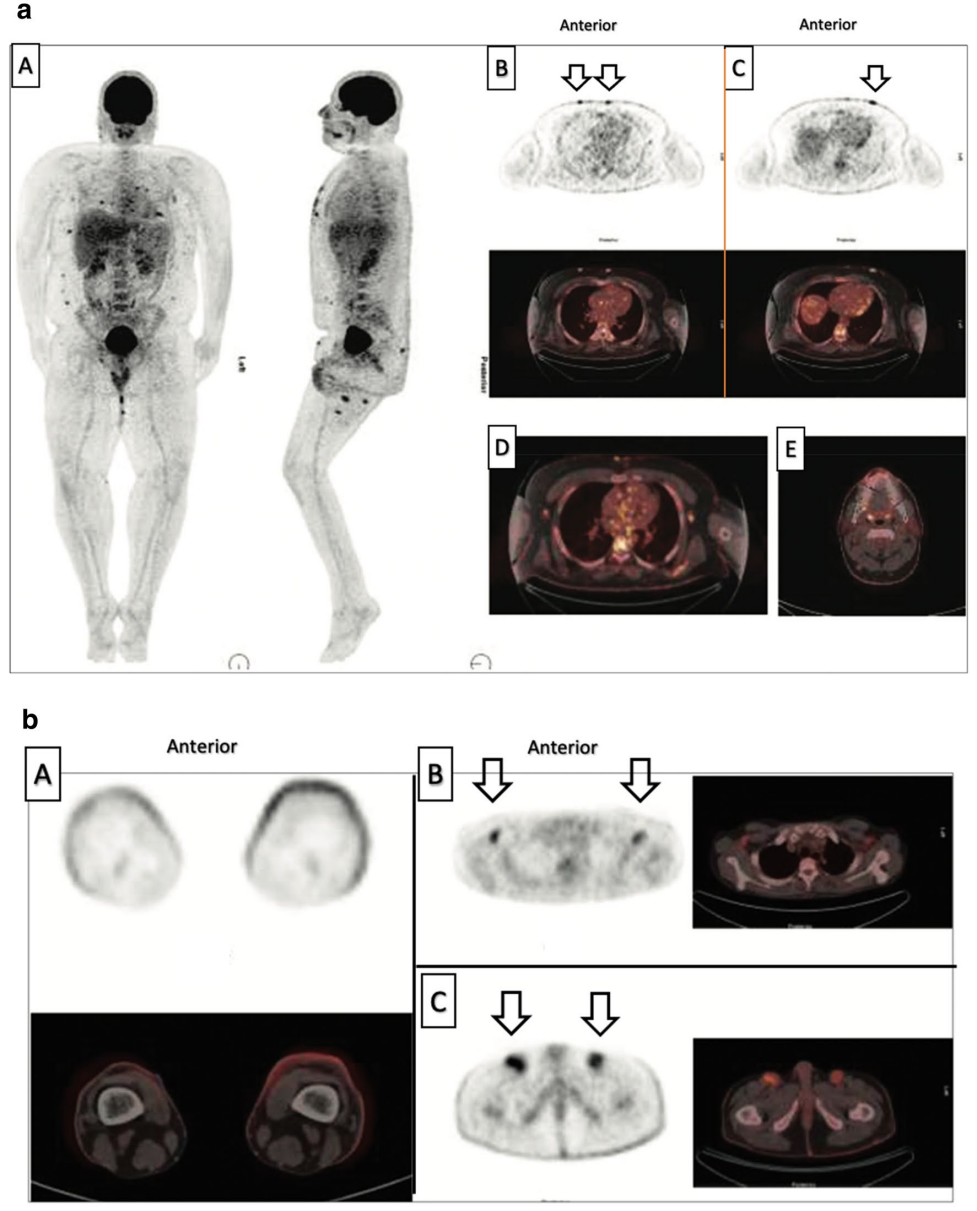
Fig. 7A. A 24-year-old male diagnosed with mycosis fungoides. AC ^18^F-FDG PET/CT three-dimensional maximum intensity projection image (**A**) displays multiple ^18^F-FDG-avid, hypermetabolic cutaneous lesions on the anterior chest (**B**, **D**), anterior abdomen (**C**), thighs and face (**E**). Examination of nonattenuation-corrected ^18^F-FDG PET/CT images in addition to AC PET images allowed detection of superficial cutaneous lesions not readily visible on CT, with the potential to impact disease staging and patient management. Images courtesy of Alanteri 2015 [22].  Fig. 7B. A 28-year-old female diagnosed with mycosis fungoides. Axial ^18^F-FDG PET/CT reveals increased ^18^F-FDG uptake bilaterally in the skin of the lower extremities (**A)**. Arrows indicate bilateral axial (**B**) and inguinal (**C**) lymph nodes display intense uptake of ^18^F-FDG relative to surrounding tissues, reflecting hypermetabolic activity. Images courtesy of Alanteri 2015 [22]

Biopsy of the skin and sentinel lymph nodes is performed during the initial evaluation of disease and histological analysis is considered as the gold standard for diagnosing PCL. Currently, the TNM/TNMB classification is used to help guide clinicians determine if, and how, a lymph node should be biopsied (Table 2A, B, C) [7, 9]. Lymph nodes larger than 1.5 cm tend to be classified as ‘suspicious’ and warrant further excision biopsy and analysis [7]. ^18^F-FDG PET/CT is highly sensitive for metastatic malignancy due to the fact that cancer cells metabolize glucose at faster rates than normal cells and ^18^F-FDG tracer is radiolabeled glucose. Cancer cells uptake greater quantities of ^18^F-FDG radiotracer than normal cells, yielding higher-intensity signals on PET images that allow physicians to distinguish cancerous from noncancerous tissue (Figs. 2, 7A, B). Therefore, ^18^F-FDG PET/CT is utilized for guiding biopsy of lymph node and bone marrow in patients with highly aggressive, late-stage PCLs [1, 22].

Currently, CT is the standard modality used to guide skin biopsy of PCLs. However, recent studies are advocating for more routine use of ^18^F-FDG PET/CT to guide skin biopsy in both indolent and aggressive PCL subtypes, due to its increased sensitivity for subcutaneous lesions as compared to CT alone. A study investigated the role of ^18^F-FDG PET/CT in detecting PC-ENK/T-NT, a highly aggressive PCL with mean survival < 12 months. They found that in the detection of malignant PC-ENK/T-NT skin lesions, ^18^F-FDG PET/CT is 96% sensitive and 98.6% specific, while CT/MRI are 68% sensitive and 97.9% specific. This study concluded that ^18^F-FDG PET/CT was significantly more sensitive for cutaneous PC-ENK/T-NT lesions than CT alone. They theorize that the routine use of ^18^F-FDG PET/CT for guiding skin biopsy in this subtype of PCL would accelerate definitive diagnosis, allowing for timely initiation of treatment in a disease with rapid progression and high mortality [20]. Studies by Jiang et al. and Davidson et al. support the novel idea that ^18^F-FDG PET/CT should be utilized over CT alone for guiding skin biopsy of subcutaneous-presenting, indolent PCLs [6, 19]. Jiang et al. found that SPTCL, an indolent PC-TCL that presents primarily subcutaneously, is highly ^18^F-FDG avid, making ^18^F-FDG PET/CT very helpful for guiding biopsy [6]. Davidson 2020 states that while ^18^F-FDG avidity is variable in indolent PC-MZL, ^18^F-FDG PET/CT is still more sensitive for the subcutaneous lesions than CT alone [19].

### Treatment response evaluation

The Deauville 5-point scale represents the standardized criteria used to interpret and evaluate the treatment response in lymphoma patients [14]. This scale allows the clinician to incorporate semi-quantitative analysis of ^18^F-FDG PET/CT scans into the assessment of PCL response to treatment [33, 34]. The patient’s mediastinal blood pool is used as a reference for background ^18^F-FDG tracer uptake, while the patient’s liver is used as a reference for “high” ^18^F-FDG uptake. Lesions with no ^18^F-FDG uptake are given a score of 1. Lesions with ^18^F-FDG uptake equal to mediastinal blood pool are given a score of 2, lesions with uptake less than the liver but more than the mediastinum are given a score of 3, lesions with ^18^F-FDG uptake moderately higher than the liver are scored 4 and those lesions with significantly higher uptake than the liver are scored as 5. The physician then uses these PET-based scores to label the lesion response to treatment as one of four categories: complete response (CR), partial response (PR), stable disease (SD) or progressive disease (PD). CR lesions have a Deauville score of 3 or less and display no bone marrow metastasis. PR lesions display reduced ^18^F-FDG uptake relative to previous scans and display no structural progression. SD lesions portray consistently elevated ^18^F-FDG uptake but lack disease progression. PD lesions have a Deauville score of 4–5 and display high ^18^F-FDG uptake or involve a new ^18^F-FDG avid focus [34].

The Deauville 5-point scale was designed primarily for use in extracutaneous lymphomas, so it was adopted in combination with the modified severity weighted assessment tool (mSWAT) to be applicable for assessment of PCL scans. Scores for PCL disease progression and response to treatment may be generated using the methods such as mSWAT, Deauville’s score, and ^18^F-FDG PET/CT. mSWAT is a qualitative method that uses visual assessment to estimate the percent surface area of the patient’s body that is diseased [11]. mSWAT score is weighted × 1 for patch, × 2 for plaque, and × 4 for tumor lesions. The mSWAT score is used to evaluate the response of the patient’s skin to treatment, with complete response indicating 100% clearance of lesions, partial response indicating 50–99% clearance and no new tumors, stable disease indicating < 25% or < 50% clearance of skin disease from baseline with no new tumors, and progressive disease indicating lesions with > 25% mSWAT increase in skin disease from baseline, or new tumors [8]. While technically there is no mSWAT equivalent for non-MF / Sézary syndrome PC-TCLs or PC-BCLs, the mSWAT system may be used [7, 11].

**Fig. 8:**
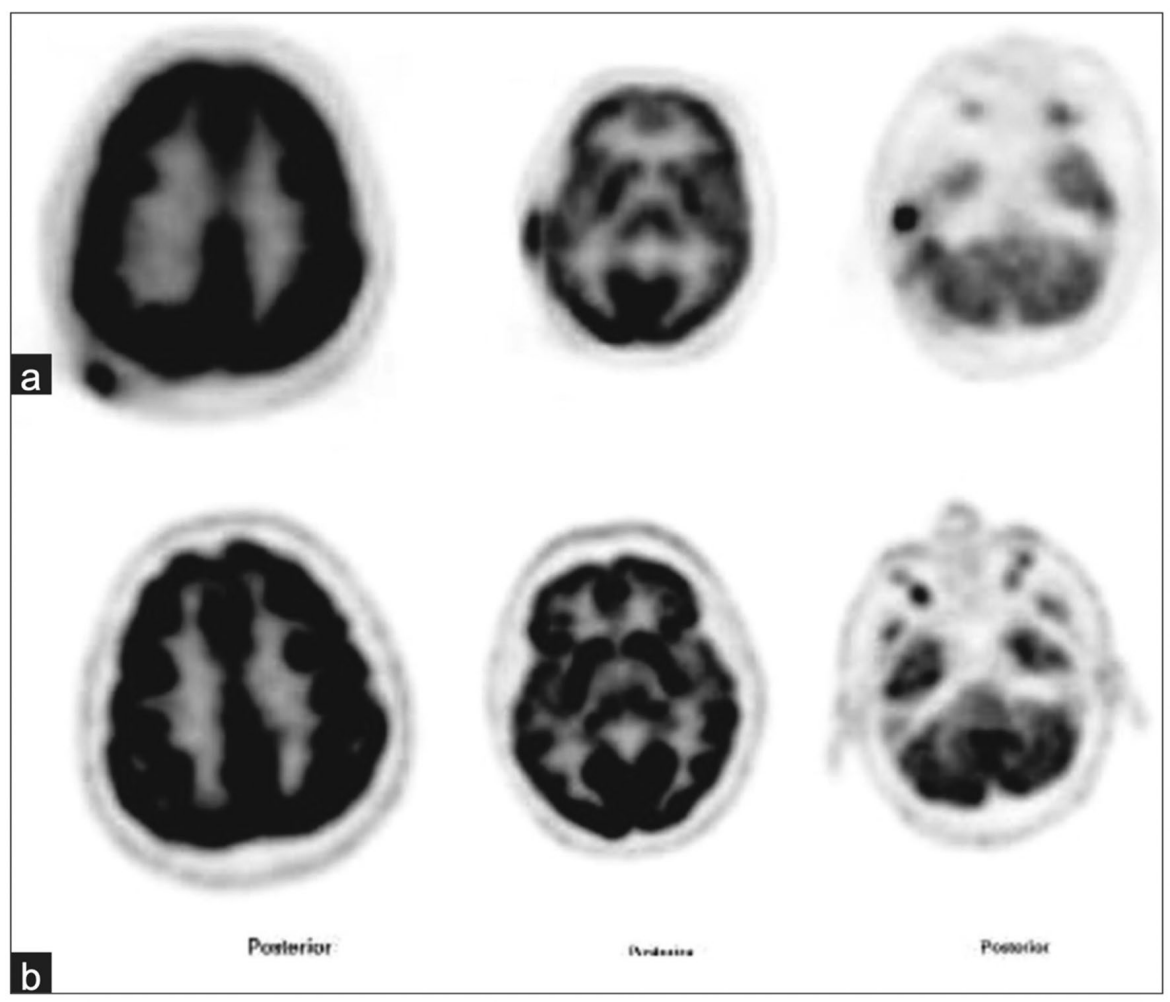
A 50-year-old male with mycosis fungoides. (**a**) ^18^F-FDG PET shows ^18^F-FDG-avid cutaneous lesions at right scalp and right external ear. (**b**) After treatment with chemotherapy, follow-up PET scan shows complete metabolic response at right scalp and right ear. Images courtesy of Alanteri 2015 [22]

Four of the nine studies reviewed in this paper investigate the utility of ^18^F-FDG PET/CT in monitoring PCL response to treatment. These studies all used a combination of the Deauville 5-point score and mSWAT to assess disease response [35]. A study by Alanteri et al. showed that ^18^F-FDG PET allows for easy visualization of disease response to treatment in cutaneous MF lesions (Fig. 8). Review of NAC PET scans improved sensitivity of ^18^F-FDG PET/CT in cutaneous MF lesions [22]. A retrospective, single-institution study by Davidson et al. concluded that ^18^F-FDG PET/CT was useful not only in initial staging but also for monitoring subcutaneous MZL lesion response to chemotherapy [19]. Subcutaneous lesions that were difficult to identify on CT appeared prominently on ^18^F-FDG PET/CT (Fig. 5). ^18^F-FDG PET/CT detection of hidden subcutaneous MZL lesions at post-treatment follow-up visits changed the disease stage in 8 patients (40%) and resulted in treatment upgrade from radiotherapy to chemotherapy in 2 patients (10%) [19]. A retrospective, single-institution study by Jiang et al. found ^18^F-FDG PET/CT to be valuable for monitoring SPTCL lesion response to treatment. Due to the subcutaneous nature of this PCL subtype and the superior sensitivity of ^18^F-FDG PET/CT for detecting subcutaneous lesions as compared to CT alone, Jiang advocates that use of ^18^F-FDG PET/CT may improve accuracy of disease burden assessment after treatment (Fig. 3) [6]. A retrospective multicenter analysis by Olszewska et al. found that while more than half of indolent PC-BCL (PC-MZL and PC-FCL) lesions are ^18^F-FDG -avid, the utility of ^18^F-FDG PET/CT in monitoring disease response to treatment is limited by the variability in ^18^F-FDG uptake in these lesions [12]. Overall, recent studies conclude that despite the variability in ^18^F-FDG uptake by indolent PC-BCL cutaneous lesions, ^18^F-FDG PET/CT continues to stage PC-BCL and PC-TCL with more accuracy than CT alone. The use of ^18^F-FDG PET/CT at post-treatment visits provides more accurate estimations of PCL disease burden, guiding providers to choose more appropriate treatments and improving the likelihood of positive patient outcomes.

One of the most recent scientific endeavors in this field is the effort to define a valid, standardized methodology by which ^18^F-FDG PET/CT may be used to calculate a semi-quantitative score of global disease in patients afflicted with PCL. ^18^F-FDG PET/CT is far superior to other imaging modalities in its sensitivity for detecting aggressive cutaneous lesions, suggesting great potential in the clinic. Several studies have attempted to delineate a methodology for using ^18^F-FDG PET/CT to generate a global disease score [32, 36–38] A singular score generated at every follow-up visit may simplify assessment of disease progression, as well as predict the prognosis of disease in patients with PCL. Although a global disease score is a potentially useful clinical parameter, the method is limited by its lack of technical standardization and external validation.

### Parameters to track disease recurrence

The recommendation for follow-up frequency may vary and is dependent on the subtype of PCL and its clinical course. Patients with stable disease or indolent PCL are recommended to follow-up with their clinical providers once every 6–12 months, as this allows for screening for disease recurrence. Patients presenting with rapidly progressive or invasive disease may follow-up for disease staging every 4–6 weeks. In addition to repeating the physical exam, peripheral blood is drawn and tested for indicators of disease such as LDH and complete blood count. Furthermore, FACS analysis of CD markers on blood cells may be used to detect disease recurrence. In the case of Sézary syndrome, a peripheral blood smear is analyzed for the presence of Sézary cells. It is recommended that patients with a history of aggressive PCL or extracutaneous disease have ^18^F-FDG PET/CT imaging performed at follow-up exams to screen for lesions not visible on physical exam [1].

### Quantification of primary cutaneous lymphoma/disease activity: global disease assessment

Studies have suggested that the generation of a global disease score representative of patient total body disease burden would be most efficient for tracking progression, recurrence, and therapeutic response of lymphomas [31, 32, 39, 40]. A singular global disease score would be calculated at the patient’s first clinical visit. This score would be re-calculated at subsequent follow-up visits; a reduced global disease score at subsequent visits would correlate directly with a reduction in the patient’s total disease burden, while an elevated score would indicate increased burden of disease (progressive disease/no-response to treatment). Having a singular score representative of patient disease would facilitate the clinician’s ability to determine the course of a patient’s disease accurately and rapidly, and then utilize the most appropriate interventions. Early and accurate staging is extremely important in patients with PCL because treatment type and effectiveness varies greatly based on the stage of disease. Global disease assessments have been shown to provide prognostic information in several other cancers [41–43]. Creating objective standards for global disease assessment among institutions can help standardize patient care and augment collaborative efforts. The methodology has previously been applied in clinical studies [44, 45], but despite its obvious advantage it has not been widely adopted in clinical practice until now.

A semi-quantitative approach to ^18^F-FDG PET/CT would permit generation of a global disease score that is highly accurate and reflective of minute changes in patient disease progression. This score would be applicable for both staging and monitoring of disease progression throughout treatment. Standardized uptake value (SUV) is a semi-quantitative measure representing the concentration of ^18^F-FDG within the volume of interest (VOI), normalized to the injected radioactivity per unit body weight, and corrected for physical decay [46]. SUV is the most used parameter to quantify metabolic activity in ^18^F-FDG PET/CT, and maximum SUV (SUV_max_) has been frequently used as a quantitative parameter. Initial studies in patients with Hodgkin’s lymphoma showed that changes in SUV_max_ were more accurate predictors of patient outcomes than changes in the Deauville score [31, 33]. However, because most lymphoma lesions are heterogeneous, the SUV_max_ is not an ideal measure of tumor metabolic activity as it refers to the voxel with highest uptake within a region of interest (ROI), and hence does not necessarily give an accurate value representative of the disease activity within a region or volume of interest. Several studies have suggested using the mean SUV (SUV_mean_) instead of the SUV_max_ to better represent a lesion’s metabolic activity because SUV_mean_ accounts for ROI heterogeneity. Additionally, it was noted that SUV_mean_ values were more susceptible to the partial volume effect (PVE), wherein the limited spatial resolution of PET causes blurring of three-dimensional images and underestimation of ^18^F-FDG tracer uptake [47]. Lesions that are moving during imaging (for example those on the heart or lung), or lesions that are smaller than the reconstructed spatial resolution (< 1.5–2 cm) are most significantly impacted by PVE, with ^18^F-FDG uptake being significantly underestimated [47]. Therefore, it is important to perform partial volume correction (PVC) on SUV_mean_ measurements. Metabolic-based volumetric parameters such as metabolic tumor volume (MTV) may be obtained using a threshold to delineate lesion activity [48]. Multiplying the MTV by SUV_mean_ of each lesion provides the total lesion glycolysis (TLG) score for each lesion. Semi-automated software enables the quantification of the SUV_max_, pvcSUV_mean_ and MTV for each ROI measured. The MTV or TLG for all lesions in the body may be summed to generate a singular global disease score representative of the total disease burden within the patient’s body.

It is important to note that a criticism of ^18^F-FDG-PET/CT is its limited ability to detect superficial lesions. However, recent studies propose that analysis of NAC ^18^F-FDG PET/CT images may allow for identification of cutaneous lesions otherwise not accounted for by analysis of AC PET images alone [22, 28, 30, 49–52]. Analysis of NAC ^18^F-FDG PET images alongside AC PET may be necessary to ensure the generation of a global disease score representative of both visceral and cutaneous aspects of total body disease burden (Fig. 6) [22, 28, 30].

### Total body PET

Until recently, PET imaging could only be performed on one region of the body at a time. The introduction of whole-body PET systems within the last 2 years has revolutionized clinical approach to staging and monitoring systemic and diffuse diseases. Whole-body PET instruments are not only able to image the entire body in one setting but also up to 68 times more sensitive than conventional PET/CT scanners. The high sensitivity of the whole-body PET allows for decreased dose utilization of radioactive tracer and reduces overall radiation exposure to the patient [47]. Furthermore, it has been proposed that the ideal time point at which ^18^F-FDG PET/CT imaging should be performed is 2–5 h post-injection [27, 53–57]. This is due to the fact that ^18^F-FDG continues to accumulate in cells over time and reaches a plateau after 3–5 h; during this time, background tracer also clears, creating an opportune moment to capture a clear image of ^18^F-FDG uptake in malignant tissues [58]. Due to the increased sensitivity of whole-body PET instruments, a longer duration between tracer administration and image capture does not reduce image quality as it might in conventional PET instruments; whole-body PET can detect ^18^F-tracers up to 12 h post-injection. This ability to acquire delayed images is important as it optimizes detection of all lesions, including those only detectable after the conventional 1-h uptake time.

## Conclusion

Studies reviewed in this paper (Table 3) conclude that the unparalleled sensitivity of ^18^F-FDG PET/CT for nodal and visceral malignancy makes it a vital tool to accurately determine disease extent, burden, and activity in patients with PCL. The high sensitivity of ^18^F-FDG tracer for metabolically active skin lesions suggests that ^18^F-FDG PET/CT may prove useful in guiding skin biopsy in these patients. These studies also conclude that AC ^18^F-FDG PET/CT is equivalent, if not superior, to CT in detecting cutaneous and subcutaneous malignancy. However, limitations of these studies make this conclusion incomplete. These studies performed analysis of the AC PET images; however, in all but one study, their methodology appeared to lack review of NAC PET images for cutaneous lesions that become undiscernible after attenuation correction. Review of the NAC PET images may significantly improve the sensitivity of ^18^F-FDG PET/CT for cutaneous malignancy, which can help further obtain a more accurate global disease score calculation.

Primary cutaneous lymphomas are rare malignancies that often go undiagnosed until they have progressed to later stages. Accurate staging of these malignancies early in the disease process is crucial to improving disease prognosis. The diagnosis, staging, treatment, and monitoring of disease in these patients is complex, requiring a multidisciplinary team composed of dermatologists, pathologists, radiation oncologists, nuclear medicine radiologists and hematologists. A standardized method for calculating a global disease score in these patients has yet to be agreed upon; such a score would facilitate accurate discernment of disease progression and allow clinicians to choose more appropriate personalized, patient-centered interventions.

## Data Availability

The datasets used and/or analyzed during the current study are available from the corresponding author on reasonable request.

## Abbreviations

AC: Attenuation-corrected
BMB: Bone marrow biopsy
CD: Cluster of differentiation
*CD4* + *PCSM-TCL*: CD4 + primary cutaneous small/medium-sized pleomorphic *T*-cell lymphoma
CR: Complete response
CSMs: CT, MRI and biopsy
CT: Computed tomography
CXR: Chest X-ray
EBV MCU: Epstein–Barr positive mucocutaneous ulcers
ESMO: European society for medical oncology
FACS: Fluorescence-activated cell sorting
^18^F-FDG PET: ^18^F-fluorodeoxyglucose positron emission tomography
^18^F-FDG PET/CT: ^18^F-fluorodeoxyglucose positron emission tomography–computed tomography
FMF: Folliculotropic mycosis fungoides
HRUS: High-resolution ultrasonography
HL: Hodgkin’s lymphoma
IPI: International prognostic index
ISL: International society for cutaneous lymphomas
LDH: Lactate dehydrogenase
LN: Lymph node
Lyp: Lymphomatoid papulosis
MF: Mycosis fungoides
MZL: Marginal zone lymphoma
MRI: Magnetic resonance imaging
mSWAT: Severity weighted assessment tool
MTV: Metabolic tumor volume
NAC: Nonattenuation-corrected
non-MF/SS: Non-mycosis fungoides/Sézary syndrome
NHL: Non-Hodgkin’s lymphoma
PC-ALCL: Primary cutaneous anaplastic large-cell lymphoma
PC-BCL: Primary cutaneous *B*-cell lymphomas
CD30(+) TCLPD: Cluster of differentiation (CD) 30(+) T-cell lymphoproliferative disorders
PC-DLBCL-leg type: Primary cutaneous diffuse large *B*-cell lymphoma* leg type*
PC-ENK/T-NT: Primary cutaneous extranodal natural killer/ *T*-cell lymphoma, nasal type
PC-FCL: Primary cutaneous follicular center lymphoma
PCL: Primary cutaneous lymphoma
PC-MZL: Primary cutaneous marginal zone lymphoma
PC-TCL: Primary cutaneous *T*-cell lymphomas
PD: Progressive disease
PET: Positron emission tomography
PR: Partial response
PVC: Partial volume correction
PVE: Partial volume effect
ROI: Region of interest
SD: Stable disease
SUV: Standardized uptake value
SUV_max_: Maximum standard uptake value
SUV_mean_: Mean standard uptake value
SPTCL: Subcutaneous panniculitis-like *T*-cell lymphoma
TLG: Total lesion glycolysis
TNMB: Tumor–node–metastasis–blood
TNM: Tumor–node–metastasis
VOI: Volume of interest
WHO-EORTC: World Health Organization–European Organization for Research and Treatment of Cancer
